# Usefulness of a Darwinian System in a Biotechnological Application: Evolution of Optical Window Fluorescent Protein Variants under Selective Pressure

**DOI:** 10.1371/journal.pone.0107069

**Published:** 2014-09-05

**Authors:** Ulrike Schoetz, Nikolaos C. Deliolanis, David Ng, Jutta Pauli, Ute Resch-Genger, Enrico Kühn, Steffen Heuer, Wolfgang Beisker, Reinhard W. Köster, Horst Zitzelsberger, Randolph B Caldwell

**Affiliations:** 1 Department of Radiation Sciences - Research Unit Radiation Cytogenetics, Helmholtz Center Munich - German Research Center for Environmental Health (GmbH), Munich, Germany; 2 Institute for Biological and Medical Imaging, Helmholtz Center Munich - German Research Center for Environmental Health (GmbH), Munich, Germany; 3 Research Group Cellular Dynamics, Max Planck Institute of Neurobiology, Martinsried, Germany; 4 BAM Federal Institute for Materials Research and Testing, Division Biophotonics, Berlin, Germany; 5 Institute of Developmental Genetics, Helmholtz Center Munich - German Research Center for Environmental Health (GmbH), Munich, Germany; 6 Institute for Toxicology, Helmholtz Center Munich - German Research Center for Environmental Health (GmbH), Munich, Germany; Chang Gung University, Taiwan

## Abstract

With rare exceptions, natural evolution is an extremely slow process. One particularly striking exception in the case of protein evolution is in the natural production of antibodies. Developing B cells activate and diversify their immunoglobulin (Ig) genes by recombination, gene conversion (GC) and somatic hypermutation (SHM). Iterative cycles of hypermutation and selection continue until antibodies of high antigen binding specificity emerge (affinity maturation). The avian B cell line DT40, a cell line which is highly amenable to genetic manipulation and exhibits a high rate of targeted integration, utilizes both GC and SHM. Targeting the DT40's diversification machinery onto transgenes of interest inserted into the Ig loci and coupling selective pressure based on the desired outcome mimics evolution. Here we further demonstrate the usefulness of this platform technology by selectively pressuring a large shift in the spectral properties of the fluorescent protein eqFP615 into the highly stable and advanced optical imaging expediting fluorescent protein Amrose. The method is advantageous as it is time and cost effective and no prior knowledge of the outcome protein's structure is necessary. Amrose was evolved to have high excitation at 633 nm and excitation/emission into the far-red, which is optimal for whole-body and deep tissue imaging as we demonstrate in the zebrafish and mouse model.

## Introduction

Fluorescent protein (FP) technology has become a ubiquitous research tool and with ever expanding applications, the need for new and improved FPs displaying unique features and specific spectral properties is growing. For intravital and whole-body imaging applications, the truly sought after FPs are those absorbing/emitting nearer and into the infrared (IR) spectrum [1]–[2]. For optimum usability, a FP's spectrum must reside in the window where tissue penetration by light is at a maximum and absorption by hemoglobin, tissue chromophores and water are at a minimum. The optical window wherein there is less light absorption and scattering lies between 600 nm and 1200 nm [3]. More precisely, light attenuation in the 590–630 nm range drops 400 times for 8 mm of tissue [4], making FPs with strong absorbance at ≥630 nm of particular interest. Further attenuating the window of opportunity is the variation in tissue densities effectively giving an upper limit in the 930–1000 nm range after which absorption by lipid and water become interfering factors [5]. For advanced imaging applications such as optical fluorescence tomography and photoacoustic (or optoacoustic) tomography the effective diagnostic and therapeutic window is in the 650–900 nm range [6].

To cope with the demand for FPs, sophisticated methods have been developed using structural data to plan site-specific mutations alone or combined with random mutagenesis. While site-specific mutation has demonstrated success, it requires extensive prior knowledge of the structure-function relationship and also requires time consuming and costly validation for every possible combination. On the other hand, the use of directed mutational activity has long been a tool of protein evolution whether e.g. it is *in vitro* via ribosomal display or *in vivo* via the use of bacterial mutator strains or via an *in vitro/in vivo* combination of error prone PCR followed by phage display. However, the standard prokaryotic-based technologies lack the post translational modifications often necessary for desired function in eukaryotic systems which led to the yeast-display methodology [7]. These methods are also associated with high costs and extensive hands-on time, which is a limiting factor for many laboratories. However, cost effective platforms exist which help to evolve proteins by introducing random mutations iteratively. In such an evolving environment, applying “rationally designed” pressure would lead to selection of the “desired result” without the need for prior knowledge of the final protein structure.

Developing B cells use somatic hypermutation (SHM) to diversify their immunoglobulin (Ig) genes. Iterative cycles of hypermutation and selection continue until antibodies of high antigen binding specificity emerge. The principle of SHM was applied to evolve mPlum from mRFP1.2 by virally introducing a randomly inserted precursor into the RAMOS cell line, and selecting via a “ratio sorting” method [8]. However, like most vertebrate cell lines, target sequences are inserted randomly and stable mutation and expression of the transcript cannot be controlled for.

The chicken B cell line DT40 is amenable to genetic manipulation and is highly recombination-proficient with a targeted-to-random integration ratio of greater than 1∶2 [9]. A DT40 mutant lacking its pseudo-Ig genes has a naturally strong hypermutation activity at the Ig light chain locus [10]. This activity can be directed to any gene of interest that can be inserted into the Ig locus [11]. The added ability to multiply and stably target the genome of DT40 with interaction partners and reporters allows for greater flexibility in not only the complexity of protein evolution, but can extend selection schemes into functional studies in cell. The use of DT40 as a protein evolution platform was demonstrated with the targeted improvement of enhanced green fluorescent protein (eGFP) producing variants displaying relative brightness up to 2.5 fold higher than the precursor eGFP, but without a spectral shift [12].

The impressive results of both labs [8], [12] in either shifting the emission or increasing the brightness illustrate that *in situ* iterative SHM has great potential. However, the evolution of advanced protein mutants with desired features is strongly dependent on efficiently targeting the gene of interest, the type of selective pressure chosen and the selection scheme allowing recovery of enhanced mutants. By choosing a very stringent readout system, we demonstrate here that a large spectral shift in both excitation and emission (Ex and Em) could be achieved with a FP using exclusively selective pressure applied on the DT40 evolution platform. We evolved a variant of the wild type dimeric FP eqFP578 [13] denoted eqFP615 (Evrogen JSC) into the FP Amrose variants with 590–594 nm/639–644 nm Ex/Em peaks as purified protein. While the spectral shifts of the peaks are significant, the more impressive attributes are the broadening of the excitation and fluorescence signals, which allow absorbance and emission into the optical window applied for whole body and deep tissue imaging. Amrose (a portmanteau of American rose) remains with ∼30% of the peak maximum highly excitable at 633 nm, an often used excitation wavelength of optical-imaging instruments, and when expressed in DT40 demonstrates functionality in the whole-mouse imaging model at 670 nm excitation.

## Results

### Development of the Amrose variants

Our strategy was to create a self-directed system which is able to deliver novel mutations without prior knowledge of where these mutations need to occur. Darwinian systems unite these advantages, as they keep a steady, ongoing evolution and “survival” is based on the selection of mutations benefiting, including not harming, the desired trait. DT40 has with 1.3×10^−5^ mutations/bp/generation [14] a high and stable mutation rate at the immunoglobulin light chain locus, guaranteeing constant diversification of any gene cloned into this region. The doubling time of the wild type cells is 10 hours [9]. Enormously diverse libraries of a gene of interest are continuously generated making the system attractive for large scale multifaceted screening.

**Figure 1:**
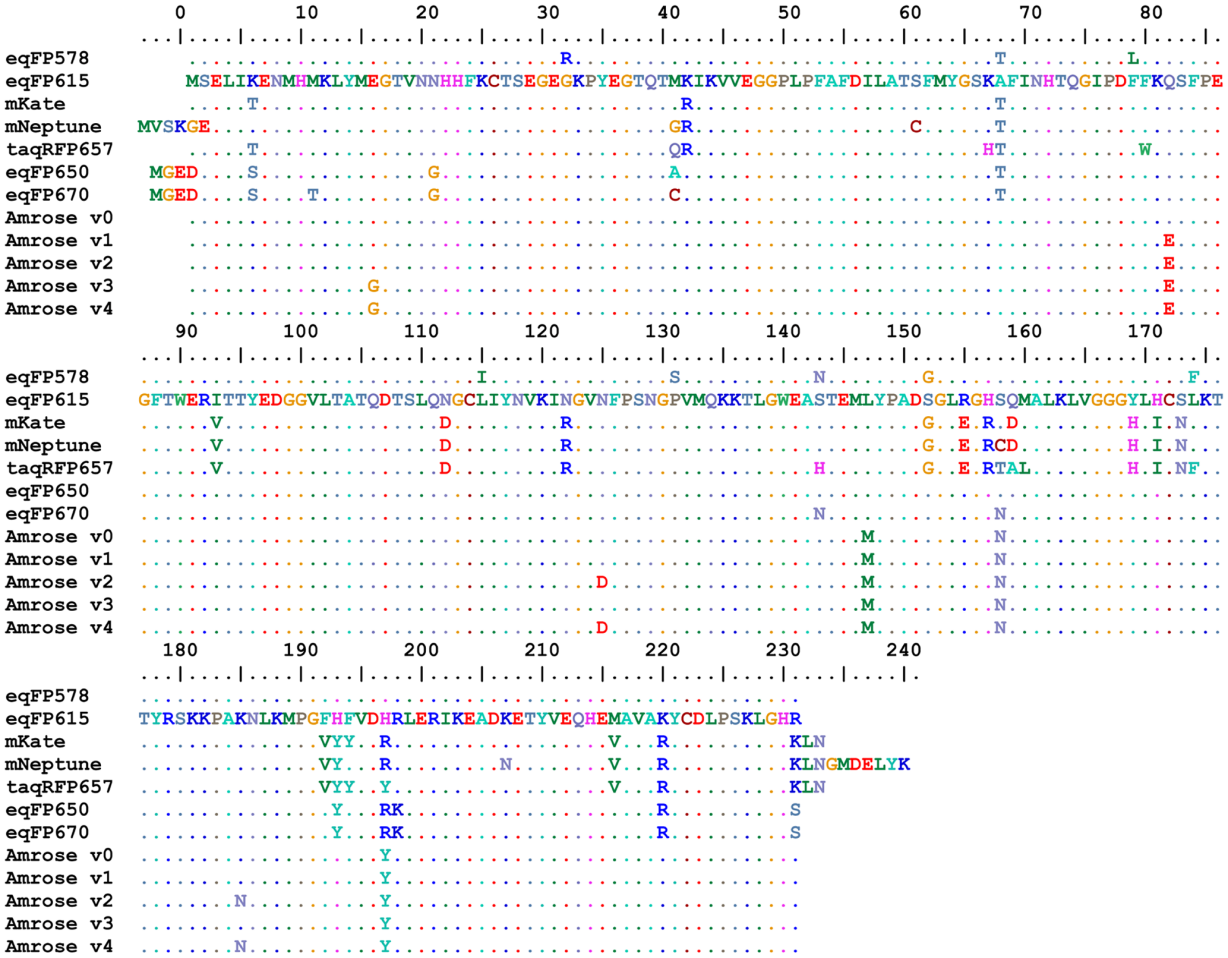
Sequence comparison. Sequence comparison (BioEdit) of selected published variants of the original eqFP578 to that of the five Amrose variants (v0-v4).

The precursor FP used, eqFP615, was transfected into the DT40 cell in a targeting construct that site specifically integrates by homologous recombination as previously described [12]. In order to put selective pressure on the highly diverse bulk population, we developed a stepwise sorting strategy that was designed to select exclusively for the stable and/or improved phenotype at each step without the explicit need to test the genotype. This allows for low-cost high-throughput screening of an ever expanding population. With each preparative sorting step, we screened 1–3×10^8^ cells corresponding to 20–25 generations and selecting those that met our criteria - approximately 0.2%. After 20 rounds of preparative cell sorts, the mutational machinery was stopped by inducing the excision of the floxed *AID* gene which is necessary for SHM [10]. A further two rounds of cell sorts to stabilize the phenotype were carried out prior to sub-cloning the bulk population and sampling 96 clones. Of the original 96 colonies picked, 78 of the surviving colonies were confirmed to be *AID* negative. Preliminary FACs screening was performed to remove obvious multiple-cell derived clones which led to the selection of 45 clones for sequencing. Analysis of the sequences identified 23 clones that were most likely single cell derived e.g. no ambiguous base calls. The 23 clones harbored three mutation patterns identified as variant 1 (12x), variant 2 (5x) and variant 3 (6x), or v1, v2, v3 (Figure 1).

**Figure 2:**
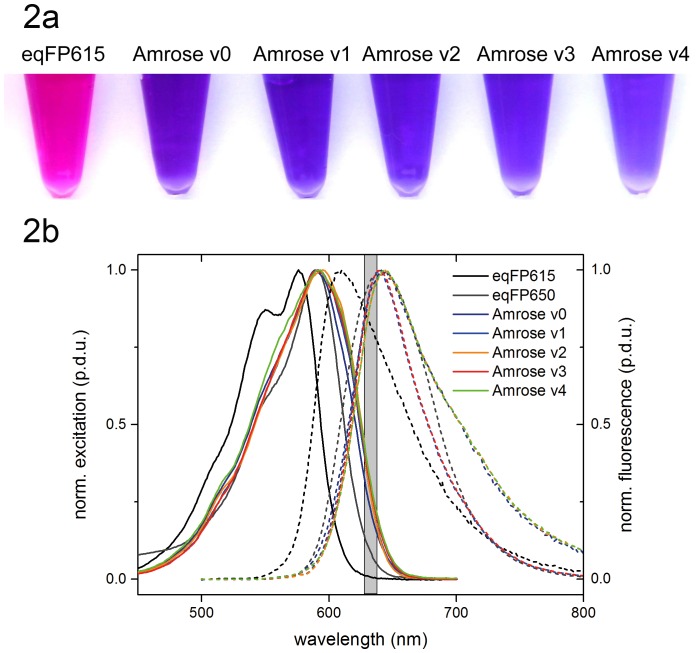
Spectral comparison. Fig. 2a. Ambient light visualization of the purified *E.coli* produced protein showing eqFP615 and Amrose variants 0–4. Fig. 2b. Normalized excitation and fluorescence spectra of qFP615 and eqFP650 and those of the Amrose v0–v4 taken from *E.coli* produced purified protein using pRSETa vector (Invitrogen). Grey bar covers the red laser line at 633±4 nm; procedure defined unit  =  p.d.u.

The relevant mutations in Amrose can be characterized via its evolutionary tree. Four consensus base mutations are shared by Amrose variants 1–3: Q82E, L147M, S158N and H197Y. Sequence sampling of sorts 13, 14 and 15 revealed that L147M, H197Y and S158N appeared by sort 13. The fourth mutation, Q82E, appeared by sort 15 and coincided with a pronounced color change in the cells under ambient lighting from more purple to more blue (Figure 2a). Variant 2 has additionally K185N and N124D which appeared following sort 15. The third major variant contains the E16G mutation which also appeared post sort 15. Joining these three variants were an engineered back mutation (E82Q) variant denoted v0 and a forward mutation variant adding v3's E16G to v2 denoted v4 (Figure 1) The reverse and forward mutations were done to further characterize the performance of the DT40 evolution system and its advantages over prokaryotic based or rational design ones. The engineered mutants were created to test one specific mutation common to all (additive) plus the combination of two sets of beneficial mutations (cumulative). Altogether for Amrose v2 for example, six mutations were introduced into the precursor eqFP615 using the DT40 evolution system. To isolate a single variant with these six specific changes in a 696 bp gene without the aid of the DT40 system and by pure random introduction, 1.1×10^17^ variants would have to be screened.

### Spectral analysis of Amrose variants

**Figure 3:**
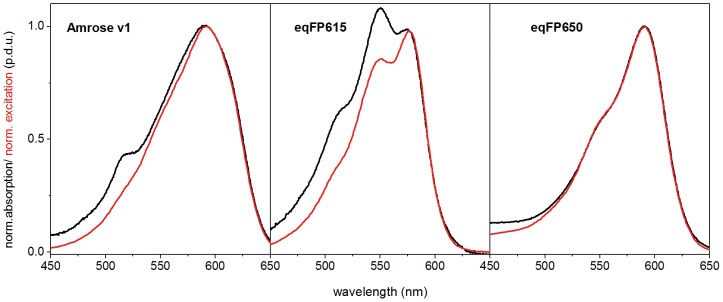
Comparing absorption and excitation. Normalized absorption and excitation spectra of Amrose v1, eqFP615 and eqFP650 taken from *E.coli* produced purified protein using pRSETa vector (Invitrogen).

Amrose v0–v4 variants, eqFP615 and eqFP650 were cloned into the pRSETa (Invitrogen) vector system to isolate fluorescent protein for analysis. The spectroscopic data obtained of purified protein in phosphate-buffered saline (PBS) are summarized in Table 1. The absorption and emission maxima of the Amrose v0–v4 variants in PBS are red shifted in comparison to eqFP615 and closely resemble the absorption and emission spectra of eqFP650 (Figure 2b, Table 1). According to the shapes of the emission spectra, the fluorescent proteins can be divided into two groups, i.e., Amrose v0, v2, and v4, and Amrose v1 and v3, respectively, with the emission spectra of the former three proteins revealing a broadening and red tail in comparison to the emission band of Amrose v1 and v3. The emission maxima, however, are nearly identical and match with that of eqFP650. A comparison of the absorption and corrected excitation spectra of the Amrose proteins suggests the presence of a certain amount of non-emissive dimers or aggregates as shown in Figure 3 exemplarily for Amrose v1, indicated by the mismatch of the signals on their short wavelength side. Dimerization seems to be enhanced for eqFP615, but in the case of eqFP650, the absorption and excitation spectrum match indicating a lower tendency to form non-fluorescent dimers. The excitation signals of the Amrose variants are broader than those of eqFP615 and eqFP650. The half widths of the Amrose variants 1–3 are 83 nm, those of eqFP615 and eqFP650 71 nm and 70 nm. Similar effects can be observed for the emission signal of Amrose 2, but not for Amrose 1 and 3. The half widths of Amrose 2, Amrose 1 and 3, and eqFP615/eqFP650 yield 87 nm, 67 nm and 78 nm, respectively. The spectral shift of the peaks together with the excitation and emission spectrum broadening allows for significant excitation into the optical window for intravital and whole-body imaging applications.

**Table 1:** Spectral properties of purified protein in PBS.

	Extinction Coefficient	Quantum Yield	Absorption Maxima/nm	Emission Maxima/nm
eqFP615	47600	0.18	551	608
eqFP650	65000r	0.12	590	641
Amrose v0	40700	0.04	590	642
Amrose v1	34800	0.02	592	638
Amrose v2	34500	0.03	594	644
Amrose v3	28000	0.02	591	640
Amrose v4	21600	0.03	594	642

r  =  reported.

Fluorescence quantum yield measurements with Amrose 0–4, eqFP615, and eqFP650 in PBS reveal quantum yields in the order of 0.02–0.03 for the Amrose proteins as compared to values of 0.18 and 0.12 found for eqFP615 and eqFP650, respectively, see Table 1. Concerning the deviations of the fluorescence quantum yield of eqFP650 from the value of 0.24 reported by Shcherbo et al. (15), we can only speculate, as the performance of these measurements including instrument calibration were not included.

Time-resolved measurements with Amrose 0–4, eqFP615, and eqFP650 in PBS reveal a tri-exponential decay behavior for the Amrose variants and bi-exponential decay kinetics for eqFP615 and eqFP650, respectively, see (Table S1). The shorter lifetimes found for the Amrose proteins correlate with their smaller fluorescence quantum yields. The fluorescence lifetimes and the quantities of the components with different lifetimes of a defined protein are independent from the used excitation wavelength.

### Validation of Amrose variants in the zebrafish model

**Figure 4:**
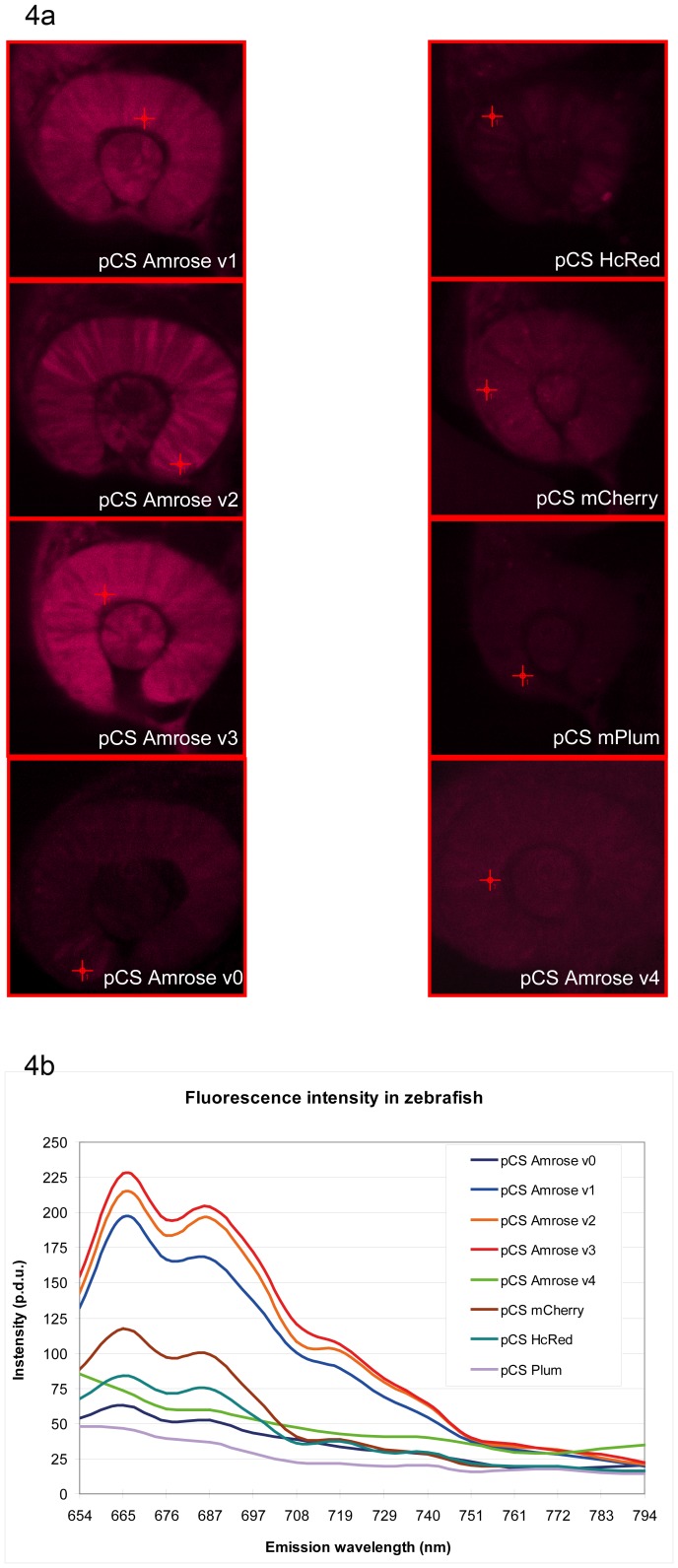
Comparison of Amrose variants in the zebrafish model. Fig. 4a. Amrose v0–v4 variants, as well as HcRed, mCherry and mPlum, visualized in zebrafish 24 hours after 100 ng/µl mRNA injection using 633 nm excitation and scanning the emission spectrum. The crosshairs indicate where the measurement took place. Fig. 4b. Intensity as recorded in zebrafish (crosshairs in Fig. 4a show where measurement took place) at an excitation wavelength of 633 nm and scanned for emission at 11 nm intervals from 654 nm to 794 nm. Procedure defined unit  =  p.d.u.

**Figure 5:**
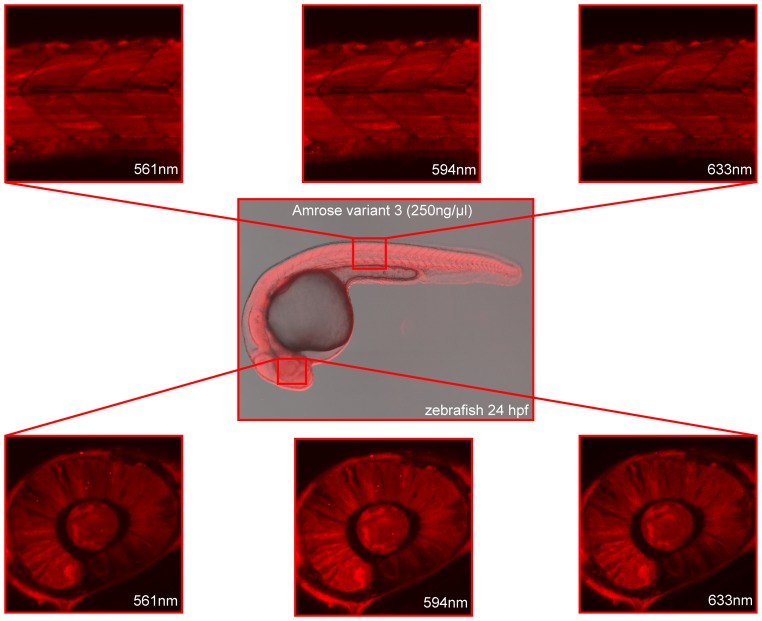
Visualization of whole zebrafish embryo at 561, 594 and 633 nm. Amrose v3 visualized in zebrafish 24 hours after 250 ng/µl mRNA injection at 561 nm, 594 nm and 633 nm excitation wavelengths. The boxes show where the measurement took place.

To demonstrate Amrose functionality in an established higher model system, Amrose v0–v4 variants were cloned into the pCS2+ vector system to synthesize mRNA by *in vitro* transcription, which was used for injection into one-cell stage zebrafish embryos. For direct comparison, equal amounts of Amrose variants 0–4, HcRed, mPlum and mCherry mRNA were used (Figure 4a, 4b) for injection (eqFP650 was unavailable at the time of this experiment). Image recording was performed with an excitation wavelength of 633 nm to scan the respective emission profiles and recording the emission in 10 nm windows from 660 to 780 nm. This revealed that Amrose variants 1–3 showed a brighter emission above 650 nm and thus in the preferred optical window for deep tissue imaging than v0, v4 or any of the three commercially available fluorescent proteins. Amrose v3, being the brightest in the zebrafish embryo (perhaps due to the lower temperature), was further analyzed. Consistent with spectroscopy measurements, Amrose v3 can be efficiently excited with the commonly used yellow (561, 594 nm) or red laser (633 nm) excitation wavelengths (Figure 5) as demonstrated here by image recording of the embryonic retina or skeletal muscle in the trunk.

In addition, *in vivo* photostability analysis was performed using three zebrafish embryos each for Amrose v3 and mCherry. The embryos were excited using a 633 nm laser set at 100% power and fast scanned producing a series of 150 pictures. The first and last picture of each set measured had ten single corresponding spots compared for loss of emission intensity with results demonstrating Amrose v3 to be comparably as stable as mCherry (Figure S1).

### Imaging of Amrose variants in the mouse model

For quantifying the performance of Amrose in deep tissue optical imaging experiments, DT40 cells expressing no FP (control), Amrose v1 and mRaspberry were used. Despite multiple attempts, we were unable to obtain cloned DT40 cells that measurably expressed Amrose v0, v4 or eqFP650. Both Amrose v1 and mRaspberry were expressed at comparable levels under the direction of the RSV promoter and in a non-mutating context (Figure S2).

**Figure 6:**
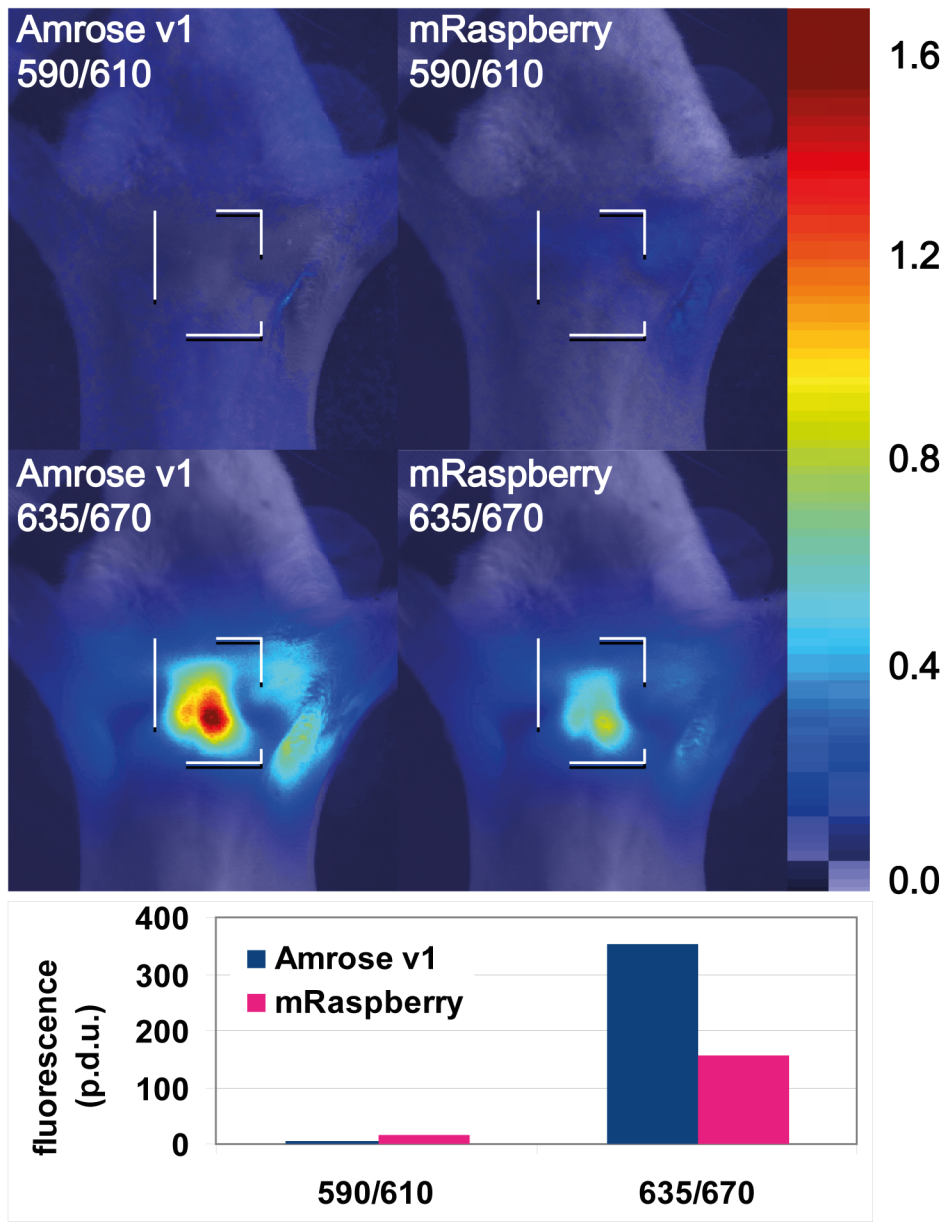
Whole-body imaging comparing Amrose v1 to mRaspberry. Fluorescence images of FPs samples in the esophagous using 2 different sets of excitation/emission wavelengths (590/610 and 635/670) overlaid on black and white images. Pixel values are in arbitrary units. Colorbar displays the transparency function. Bar chart – Comparison of accumulated FP intensity over a region of interest (dotted square) at the peak wavelength. Procedure defined unit  =  p.d.u.

Approximately one million cells of each clone were inserted into 0.9 mm capillary tubes and centrifuged to form a small pellet; this amount corresponds to a small primary tumor, or average size metastasis. The capillary tube was inserted into the esophagus of a euthanized mouse and imaged in transillumination mode mimicking the procedure designed for live mouse imaging in the near-infrared [2]. The capillary tube with the Amrose v1 expressing DT40 cells was removed and replaced with a similar tube containing non-fluorescing DT40 cells as control and mRaspberry expressing DT40 cells for comparison. In order to eliminate the tissue auto-fluorescence, the background fluorescent image from the control cells is subtracted from the corresponding Amrose v1 and mRaspberry fluorescent images to yield the net fluorescence of the FPs. The fluorescent images from the FPs for two different excitation and emission wavelengths corresponding to available laser diode wavelengths are presented (Figure 6). At 590 nm, mRaspberry is excited close at its peak wavelength, still the detected signal is marginally above the noise level and Amrose signal is below noise level and practically undetectable. The reported excitation spectra for mRaspberry at 630, although small (<10%), is still capable to excite the molecule and emit fluorescence. In addition, our own data shows that mRaspberry expressed in live DT40 cells has 28% excitation at 630 nm or roughly one third that of the Amrose variants tested. At 635 nm in the mouse model, there is a one order of magnitude increase for the mRaspberry signal and Amrose signal is increased to 2.25 times brighter than mRaspberry.

**Figure 7:**
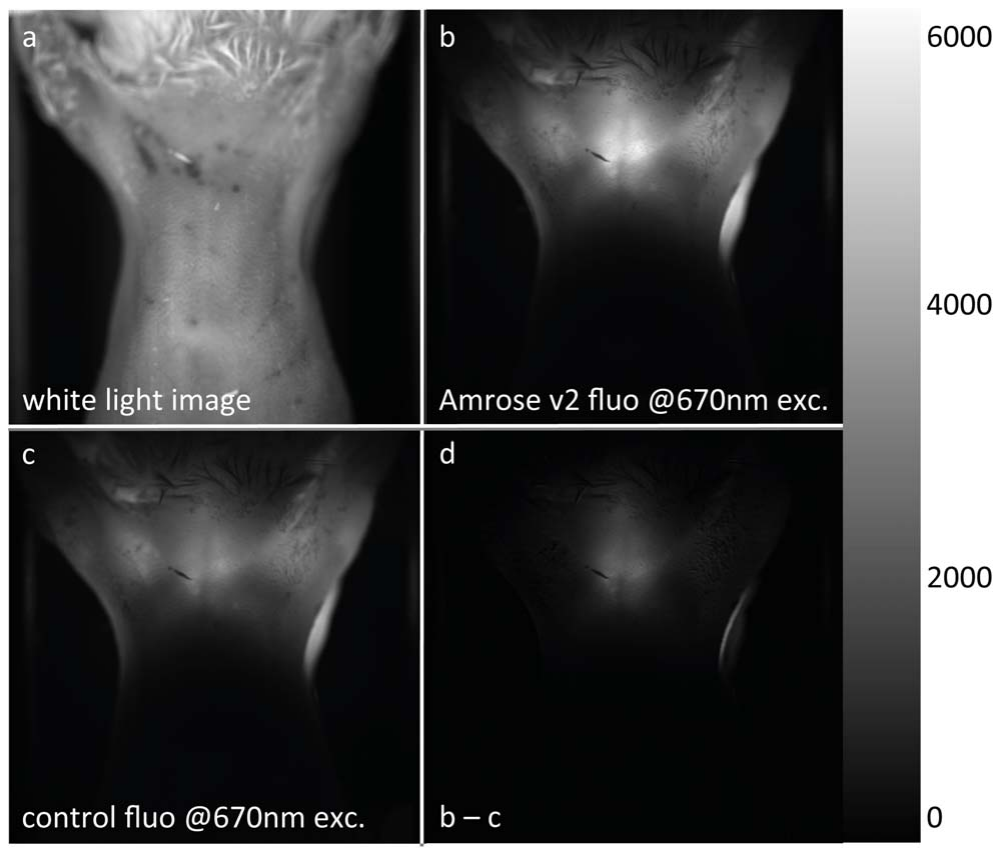
Whole-body imaging using Amrose v2 in FMT. Amrose v2 expressing DT40 cells imaged with a Fluorescence Molecular Tomography (FMT) system which allows for tomographic reconstruction of the tube using 670 nm excitation and 710 nm emission.

A similar experiment was also performed to test the feasibility of imaging Amrose in a Fluorescence Molecular Tomography (FMT) system [4] that allows the tomographic reconstruction of fluorescence bio-distribution (Figure 7). The details are as previous except that the DT40 cells used were expressing Amrose v2 and there was no available comparison FP for this wavelength. The tissue was excited by a 670 nm continuous wave laser (B&WTek, Newark, DE) and the emanating photon field is imaged at 710 nm. This clearly shows that DT40 expressed Amrose v2 can be excited at 670 nm and the emitted fluorescence can be detected through mouse tissue.

## Discussion

We demonstrate here the expanded ability of a Darwinian system to answer a need by using solely selective pressure. Applying this technology, we generated Amrose; a bright far red shifted optical imaging expediting fluorescent protein. Another research group [16] used hypermutation in a similar way in Ramos cells to evolve mRFP1.2 (excitation/emission peaks of 590/612 nm) to mPlum (590/649 nm) and mRaspberry (598/625 nm). The shifts in emission peaks are significant; however, absorption shows little to no spectral shift, presumably due to the ratio sorting protocol used at a constant excitation wavelength of 568 nm. Additionally, targeting, stability and easily stopping the mutation machinery in Ramos cells are areas of concern for an evolution platform technology. Which is perhaps why the same research group used a combination of site directed mutagenesis and error prone PCR for the later development of mNeptune [16]. Two other publications [17]–[18] have described similar achievements. Their developmental approach also involved knowledge-based mutagenesis and error prone PCR. While the DT40 platform does not appear to share the disadvantages of the Ramos cells, the product of any such platform technology is defined and limited by the selection scheme. The Amrose FP precursor, eqFP615, was derived from the dimeric eqFP578. As Amrose was selected for attributes advantageous to whole-body imaging, and absent known mutations leading to monomerization, we would suspect that the Amrose variants are able to multimerize. However, such multimerization capabilities should not interfere with the intended goal of non-toxically labeling cells for deep tissue and intravital imaging as the Amrose variants 1–3 were developed in vertebrate cells.

The three selected Amrose variants share the Q82E, L147M, S158N and H197Y mutations that make up variant 1, where the appearance of Q82E at sort 15 coincided with a pronounced color change in the DT40 cells from more purple to more blue, see v0 versus v1 in Figure 2a. Reversion of the Q82E mutation (v0) revealed it has a role in brightness and expansion of the absorption band in the eukaryotic model that would not have been revealed in the prokaryotic model (Figure 2b, 4a). Variant 2 has additionally K185N and N124D mutations and variant 3 contains the E16G mutation. The E16G appears to be a significant variation from variant 1 displaying an enhanced brightness in context of the Zebrafish model where transcript stability, post-translational eukaryotic context and/or lower temperature may play a role. Combining the mutations of v2 and v3 to give v4 resulted in a slight shift in the absorption and broadening in the excitation spectrum as determined by bacterial expression. However, v4's emission in the Zebrafish model is non-existent, similar to v0, whereas in bacterially derived pure protein it is strong and most comparable to v2 (Figure 2b, Table 1). The selected variants 1–3 perform well in the higher organisms as well as in bacterial systems, while the artificially engineered variants do not translate forward.

In eqFP578 and its derivatives, the chromophore sequence M-Y-G sits at the center of an α-helix which is located along the axis of an 11 stranded β-barrel [13]. Both sides of the barrel are closed by loop caps. The chromophore establishes hydrogen bonds with water molecules and side chains of the adjacent amino acids as well as van der Waals contacts, all of which influence its fluorescent properties. Mutations of amino acids occupying or influencing key positions could result in a spectral shift. Considering the literature, and absent crystallographic data for Amrose specific variants, we speculate that Q82E and N125D in combination with K185N and E16G have either a role in the cis-trans isomerization profile, chromophore packing or influence hydrogen bonds or van der Waals contacts as described for eqFP578, Katushka [19] and mNeptune [16]. The pH dependent cis-trans configuration can switch an FP from a fluorescent to a non-fluorescent state. This involves amongst other amino acids Ser158 and Arg197 where the Amrose variants have the S158N and H197Y mutations. The recently described eqFP670 and TagRFP657 [15], [18] share these key mutations that were both selected for in Amrose; S158N (eqFP670) and H197Y (TagRFP657). Lin et al. [17] performed saturation mutagenesis at position 197 and came to differing results in regards to having a tyrosine at this position when cumulatively combined with mNeptune's other mutations which suggests context matters. In the eqFP611 [20] variant, the hydroxyphenyl group of the chromophore was described as being in the trans-configuration and surrounded by F174 and H197 [21]. Histidine and phenylalanine make van der Waals contacts with S158 determining the orientation of the hydroxyphenyl ring and influencing the color. For example, DsRed is packed more loosely in this area and thus the color is not in the infra-red. Both Amrose and eqFP670, while having F174L, also have the amino acid change from serine to asparagine at position 158. Probably the presence of an additional carboxamid strengthens van der Waals contact to L174 and tightens the packing. It is of note that the DT40 evolution platform allowed the discovery of both specific amino acid mutations S158N and H197Y simultaneously via selective pressure in addition to Q82E which was revealed to be highly relevant in the eukaryotic model.

Beyond this, selection of functionally assured variants with a minimum of essential mutations is enabled. Even silent mutations (v3 contains a silent mutation) which may contribute to RNA stability, processing and epigenetic effects could be selected for in an *in cell* evolution system and that is another level that *in silico* technology is not matured enough to predict. If desired, the spectrum of mutations can be further modulated with the use of trans-acting factors such as *UNG* or *E2A* [22]–[23]. The DT40 evolution platform also supports using gene conversion competent cells and homologous donor sequences as “pseudo genes” to evolve proteins of interest [24]–[25]. Inasmuch, the value of the system is greatly benefited by opening up alternative avenues for diversification. For instance, Amrose could be cloned into the Ig locus together with other far-red proteins as potential gene conversion donor sequences to further improve or diversify Amrose's properties.

Amrose has been shown to be highly competitive with other fluorescent proteins of this class with using only a minimum of mutational changes to achieve the desired function. Although relative brightness was expected to be stronger, taking into account that Amrose excitation and emission in whole-body imaging is taking place beyond 630 nm there is a significant advantage over the shorter wavelength but brighter orange-red proteins. Taken together, the spectral shift of the peaks and the emission spectrum broadening allows for significant excitation into the optical window for intravital and whole-body imaging applications. Additionally in photoacoustic imaging, a novel imaging technology [26], the signal is generated from the absorbed energy that is not radiated in the form of fluorescence. As a consequence, high molar excitation coefficient (EC) and low quantum efficiency (QE) yield higher signal to noise ratios and is an advantage in photoacoustic imaging. Its usability in multicellular organisms has been demonstrated and it will be an effective tool for deep tissue optical imaging experiments for which it was created. As the intent is to non-toxically label live cells for whole body and deep tissue imaging, multimerization should not be an issue. Furthermore, the DT40 platform is not restricted to FPs alone as protein evolution can be achieved for any protein of interest whereby a suitable selection scheme can be implemented [11].

## Materials and Methods

### Ethics statement

All procedures involving euthanized mice (overdose of Ketamin and Xylazine) were performed ex-vivo; therefore, no ethics committee approval is required. All research on zebra fish was conducted in accordance with the EU Directive 2010/63/EU. With respect to this directive an animal protocol approved by an ethics committee is only required for freely feeding zebra fish larvae (starting from day 6 post fertilization). Our recordings were done one day after fertilization and thus do not require an approval by an ethics committee.

### Cell culture

DT40 cells [27] were cultured in chicken medium (CM; DMEM/F-12 supplemented with 10% fetal bovine serum, 1% chicken serum, 2 mM L-glutamine, 0.1 mM b-mercaptoethanol and penicillin/streptomycin) at 41°C in a 5% CO_2_ environment. To keep the cells vital, an optimal cell density of 0.5–1.5×10^6^ cells per ml medium was maintained.

*AID* expression in the DT40 clones (a prerequisite for continuous hypermutation) was assured by transfection of a floxed *AID*-*IRES* (internal ribosome entry site) -*GPT* (guanine phosphoribosyl transferase) bi-cistronic cassette. The DT40 cell clone used expresses an inducible form of the Cre recombinase (a MerCreMer fusion protein) in order to excise the floxed *AID* and thereby stop mutational activity if desired. However, leaky background activity of MerCreMer can lead to undesired excision of the floxed *AID*-GPT expression cassette during prolonged culture. To avoid the appearance of *AID* negative clones, cells were cultured in the presence of 0.5 mg/ml of mycophenolic acid for three days following each preparative FACS sort. To encourage healthy growth of the sort collected 5,000–20,000 cells, the post-sort drug treatment was performed in a single well (of a 24 well plate) containing 2 ml of the drug-infused CM. After the drug treatment, the cells were transferred first to a single well of a 6 well plate containing 5 ml CM and then to 50 ml flasks containing 20–50 ml CM in order to keep optimal cell density and avoid mass cell die-off. Preparative sorts were performed at 10–14 day intervals with 10^7^ unsorted cells being frozen for backup, 10^6^ unsorted cells being allowed to further propagate in case of contamination during the sort and 1–3×10^8^ cells being used for the cell sort.

To isolate single colony variants in a non-mutating background, the floxed *AID* expression cassette was excised by culturing the cells in CM containing 20 nM 4-hydroxitamoxifen (SIGMA) for three days followed by two rounds of cell sorting for stabilization. The resulting cell population “FR_S22” was subcloned to produce single cell mycophenolic acid sensitive colonies (*AID* excised) for sequence analysis. For subcloning, cells were dispensed onto three 96 well plates in concentrations of 300, 100 and 30 cells per plate in a volume of 200 µl CM per well. After seven to nine days of incubation, single colonies became visible and were picked by placing a pipette tip into the center of the colony, 10 µl were withdrawn and transferred to a single well of a 24 well plate containing 1 ml CM. The subclones were tested in normal CM and CM containing 0.5 µg/ml mycophenolic acid for their sensitivity to this drug. Cell clones that did not survive a 3 day mycophenolic acid treatment were assumed to be *AID* negative and candidates were further confirmed by PCR using the *AID*-specific primer-set 5′ cccagatcttgcttgtgaagtcttcttattgctg 3′/5′ cccgctagcgccaccatggacagcctcttgatgaagagga 3′.

### Flow cytometry

**Figure 8:**
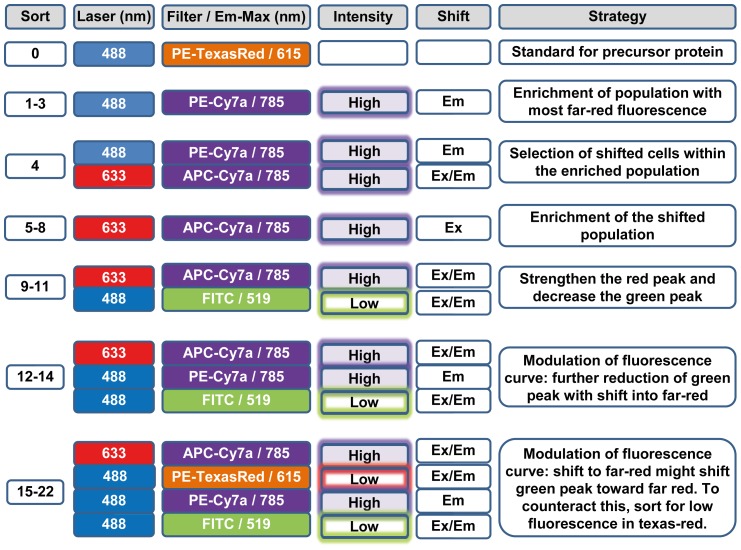
Selection criteria. Description of the laser and filters used to ideally shift a cell population into the far-red via preparative FACS sorts. Corresponding emission maxima (Em-Max) are described behind the filter. Sorting was restricted to 0.2% of the cells that best matched the sort criteria.

Fluorescent activated cell sorts were performed using the FACSAria for sort 1 through 15 and the FACSStar PLUS (both BD Biosciences) for sort 16 through 22. 1–3×10^8^ cells were spun down and resolved in 3–10 ml PBS (density of the cells was adjusted to the flow rate of the cell sorter). Selection criteria for the preparative FACS sorts were as outlined (Figure 8). The initial sorts sought variants with increased emission at 785 nm. This was followed by selection of variants capable of being sufficiently excited at 633 nm. With the DT40 system it did not take long to then be able to sort based on the double parameters of strong Ex/Em in the longer wavelength channels and weak Ex/Em in the shorter wavelength channels. Thus, achieving self-directed evolution based on the selective pressures placed on the system.

### PCR, cloning and sequencing

The cloning and Ig locus integration of eqFP615 was performed as previously described [12]. Crude genomic DNA extracts (gDNA) of 78 *AID*-negative Amrose subclones were prepared by incubation of cell pellets of app. 1.5×10^6^ cells in Expand Long Template PCR system Buffer 2 (Roche) supplemented with 0.5% Tween20 and 0.1 mg/ml Proteinase-K. Incubation was carried out at 56°C for 50 min and 95°C for 10 min. The PCR amplification of the Amrose transgenes was performed with the Expand Long Template PCR System (Roche) using 1 µl aliquots of the crude extracts and the primer pair rbc580/rbc271 (5′ gtgtgttggaggtcgctgagtagtgcgcgagc 3′/5′ ggctgattatgatcctctagagtcg 3′). The reaction mix per sample consisted of 6.33 µl H_2_O, 1 µl Buffer 1, 1 µl Cresol Red 10x, 0.2 µl dNTP mix (10 mM), 0.07 µl polymerase mix, 0.2 µl each forward/reverse primer (25 µM stock) and 1 µl template gDNA for a total reaction volume of 10 µl. PCR conditions were: 2 min of initial incubation at 93°C, 35 cycles consisting of 10 sec at 93°C, 30 sec at 65°C and 5 min (plus an added 20 sec per cycle) at 68°C and a final elongation step of 7 min at 68°C.

The amplicons were gel purified (GPU) using the QIAquick Gel Extraction Kit (Qiagen) according to manufacturer's protocol. 2 µl aliquots of the purified PCR product were used for sequencing PCR with BigDye Terminator v3.1 Cycle Sequencing Kit (Applied Biosystems). The reaction mix per sample consisted of 0.5 µl DMSO, 4.1 µl DEPC H2O, 1 µl 5x buffer BDT, 2 µl BDT, 0.4 µl primer (25 µM stock) and 2 µl template GPU. The primers used for sequencing were rbc584 (5′ agacgggtctgacatggattggacgaa 3′) and rbc276 (5′ ccgcccgggtcggcttcggtcggagccatggagatc 3′). PCR conditions were: 4 min of initial incubation at 96°C, 45 cycles consisting of 30 sec at 95°C, 20 sec at 50°C and 4 min at 60°C. To precipitate the DNA from the sequencing reaction on a 96-well PCR plate, 2.5 µl of 125 mM EDTA were pipetted into each well. A spin down at 900x gravity (g) for 2 min was performed. 30 µl of 100% ethanol were pipetted into each well and the mix was incubated for 15 min at room temperature. The plate was centrifuged at 2,000×g and 4°C for 30 min. Afterwards, the supernatant was removed by a short spin of the plate bottom up at no more than 9×g. To wash the pellet, 50 µl of 70% ethanol was pipetted into each well and again the plate was spun bottom up at no more than 9×g for 5–10 sec. The pellet was dried at 70°C for 3 min. The DNA was resuspended in 30 µl chromatography grade H_2_0 and analyzed on an ABI 3730 DNA Analyzer (Applied Biosystems).

For the back and forward mutations, targeted mutagenesis was performed using standard PCR methods. To introduce E16Q to Amrose v2, a forward primer was simply designed to introduce the change (rbc709 5′ tacgctagcgccaccATGAGCGAGCTGATCAAGGAGAACATGCACATGAAACTGTACATGG**g**GGGCACCGTGAACAACC 3′) using v2 template DNA. For the backward mutation of E82Q, overlapping primers were designed to introduce the reversion (rbc711 5′ GCATCCCCGACTTCTTTAAG**c**AGTCCTTCCCTGAGGGCTTCACATGGGAG 3′, rbc712 5′ AAGCCCTCAGGGAAGGACT**g**CTTAAAGAAGTCGGGGATGCCCTGGGTGTG 3′) creating two PCR products that were then used in ligation PCR with Amrose forward primer rbc710 5′ tacgctagcgccaccATGAGCGAGCTGATCAAGGAGAACATGCACA 3′ and reverse primer rbc664 5′ ttagatctTCATCTGTGCCCCAGTTTGCTAGGGAGGTCGC 3′. The resultant clones were sequenced for confirmation.

To ensure comparable transcription levels for the deep tissue direct comparison of mRaspberry to Amrose v1 in DT40 cells, cDNA was made from total mRNA using the RNeasy Mini Kit (Qiagen) according to manufacturer's protocol. Semi-quantitative PCR was performed as previously described [12] using the conserved forward primer for both (rbc740 5′ CCCTACGAGGGCACCCAGACC 3′) and the reverse primer rbc742 5′ GCCGTCCTGCAGGGAGGAGTCCTGGGTCACG 3′ for mRaspberry and rbc638 5′ GCCGTTCTGGAGGCTGGTGTCCTGGGTAGCG 3′ for Amrose v1. Primers for B-cell activation factor cDNA were used for control: rbc9 5′ AACTCTCCCTCTGGCTTTAG 3′ and rbc10 5′ AGACACTTGCAGGAAGGAAG 3′ (Figure S2).

### Fluorescent protein purification

For purified protein measurements, Amrose v0-v4 variants and eqFP615 were cloned into pRSETa (Invitrogen) via the BamHI-XhoI restriction sites introduced with primers rbc737 5′ taaGGATCCATGAGCGAGCTGATCAAGGAGAACATGCAC 3′ and rbc738 5′ taaCTCGAGTCATCTGTGCCCCAGTTTGCTAGGGAGGTC 3′. For eqFP650, the same was done using primers rbc771 5′ taaGGATCCATGGGAGAGGATAGCGAGCTGATCTCCGAG 3′ and rbc772 5′ taaCTCGAGTCACTGTGCCCCAGTTTGCTAGGCAGGTCGCA 3′ The resultant clones were sequenced for confirmation, then used to transform *E. coli* BL21 via heat-shock and grown to an OD600 of ∼0.6–0.8 in 200 ml LB containing 50 µg/ml ampicillin. Protein expression was induced with 1 mM isopropyl β-d-thiogalactoside. Bacteria were allowed to express the recombinant protein overnight at room temperature, followed by harvesting by centrifugation at 6000 rpm for 10 min. The pellet was resuspended in 10 ml of resuspension buffer (20 mM NaHPO4, 300 mM NaCl, 20 mM Imidazole, pH 7.8), and cell lysis was induced by freezing at −80°C with PMSF, Leupeptin and Pepstatin added to inhibit protease activity. The mixture was sonicated on ice with 0.1% Triton X-100 for 30 min, followed by centrifugation at 13,000 rpm for 30 min. The supernatant was mixed with 300 µl of Ni-NTA agarose (Qiagen), and the proteins were allowed to bind for 3 hours, followed by washing with 20 ml of resuspension buffer, and eluted with 500 µl of elution buffer (20 mM NaHPO4, 300 mM NaCl, 10% glycerol, 150 mM Imidazole, pH 7.8). Protein concentrations were determined with the QuickStart Bradford Protein Assay kit (Bio-Rad) after protein denaturation with 3 M Guanidine HCl at 95°C for 3 minutes, using the Ultrospec3000 spectrophotometer (Pharmacia Biotech).

### Optical properties analysis

The Beer-Lambert law (A = ϵcl) was used to calculate the extinction coefficient (EC). Briefly, protein concentrations were determined using the Bradford Assay and absorption was recorded on the Ultrospec3000 spectrophotometer. Calibration was done using purified BSA as a standard (NEB). Protein samples were diluted to the linear range of the assay, the weight of protein/ml was calculated in triplicate and the molarity was calculated using the known weights of the proteins.

The absorption spectra were recorded on a CARY 5000 spectrophotometer (Varian Inc., Palo Alto, USA). Fluorescence measurements were performed with the calibrated spectrofluorometer FSP920 (Edinburgh instruments) equipped with Glan Thompson polarizers in the excitation and the emission channel (set to 0° and 54.7) [28]. Typically, the emission and absorption spectra of the solutions were determined in 4 mm cuvettes (Hellma, type 108F-QS) in duplicate. The samples were excited at 525 nm for eqFP615 and 565 nm in the case of the Amrose variants and eqFP650, always using absorbance's of 0.02 to 0.06 to minimize inner filter effects and reabsorption. Rhodamine 101 in ethanol was used as fluorescence quantum yield standard for both excitation conditions. The excitation spectra of all samples were recorded at emission wavelength of 610 nm for eqFP615 and in the case of the Amrose variants and eqFP650 at 645 nm, respectively, and corrected for the wavelength-dependent spectral photon flux of the excitation channel [28]. These excitation spectra represent the absorption spectra of the emitting species.

The fluorescence quantum yield values (Φ_f_) of the fluorescent protein were calculated from integrated, blank, and spectrally corrected emission spectra relative to the standard Rhodamine 101 solved in ethanol (Φ_f_ = 0.915) [29] using the following formula [30].



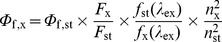
(eq. 1)


Here, *f(λ*_ex_ *)* is the absorption factor at the excitation wavelength *λ*_ex_, *F* the integral emission intensity, i.e., the area under the blank and spectrally corrected emission spectrum on a wavelength scale, and *n* the refractive index of the solvent(s) used. The subscripts *x* and *st* denote sample and standard respectively. Typical instrument-related relative measurement uncertainties as derived from previous experiments are less than ±6% (for 0.9>Φ_f_>0.1) [31].

Time resolved fluorescence measurements were performed at three different excitation wavelengths, i.e., 525, 565, and 595 nm, respectively and read out at 645 nm for the Amrose variants and eqFP650 with the time-resolved fluorometer FLS 920 from Edinburgh Instruments. The eqFP615 was excited at 525, 550 and 575 nm and the emission monitored at 605 nm. The instrument response function (IRF) was measured and monitored at the respective excitation wavelengths using a scattering solution. The different decay components were obtained after deconvolution with the IRF from a triple or double exponential fit.



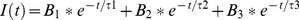
(eq. 2)


### mRNA synthesis, injection and zebrafish image recording

Selected Amrose variant fluorescent proteins were subcloned into the pCS2+ expression vector [32] (EcoRI-XbaI) using the gel purified amplicons generated above. The resultant clones were sequenced for confirmation. NotI-templates were generated for mRNA transcription using the SP6 mMessageMachine Kit (Ambion) according to the manufacturer's protocol. After purification with RNeasy (Qiagen) columns, mRNA was injected at either 100 ng/µl or 250 ng/µl into one-cell stage embryos, which were raised in 30% Danieua/0.15 mM phenylthiourea (PTU). At 24 hours post fertilization (hpf), fluorescent embryos were dechorionated, mounted on coverslips in 1.2% ultra-low gelling agarose, overlaid with 30% Danieua/PTU and analyzed by laser scanning confocal microscopy (Zeiss LSM510 Meta).

### Deep tissue imaging

The samples were excited with a NdYAG-pumped wavelength-tunable dye laser (Sirah Laser, Kaarst, Germany) and imaged with a f#1.2 50 mm lens (Nikkor, Japan) and a low-noise cooled 512×512 16-bit CCD camera (VersArray, Princeton Instruments, Acton MA), using 10 nm FWHM band-pass interference filters (Chroma, Bellows Falls, VT) centered at 610, 670 and 710 nm. Imaging was performed in a dark room. Approximately 1 million cells expressing Amrose variants and mRaspberry, as well as control cells, were inserted in the bottom of a small capillary tube with 0.9 mm diameter and centrifuged to occupy approximately 3 µL of volume. First, the tubes were imaged in reflectance mode against a dark background for a series of excitation wavelengths and emission filters to establish a baseline of fluorescent protein expression. Following, the tubes were inserted in the esophagus of a euthanized mouse with the cells placed above the heart. The mouse tissue was illuminated at the dorsal side and the emerging photons were imaged from the ventral side in transillumination mode. The total tissue thickness from illumination point to chest was approximately 1.5 cm with the esophagus being approximately in the mid-distance. For all images acquired the camera dark noise was subtracted and the pixel counts were normalized for exposure time (5 sec), laser intensity (20–200 mW), and transmission of interference filters. The auto-fluorescence images were subtracted from fluorescence to get the net fluorescence. The fluorescent images were overlaid on background white-light images using a variable transparency function. The intensity of the fluorescent proteins was measured by summing up the pixel values in a selected region of interest in the area on the maximum signal.

## Supporting Information

Figure S1 **Photostability of Amrose compared to mCherry.** Photostability analysis was performed using three zebrafish embryos each for Amrose v3 and mCherry. The embryos were excited using a 633 nm laser set at 100% power and fast scanned producing a series of 150 pictures. The first and last picture of each set measured had ten single corresponding spots compared for loss of emission intensity.
(TIF)

Figure S2 **Semi-quantitative expression analysis of Amrose compared to mRaspberry.** Amrose and mRaspberry were expressed at comparable levels under the direction of the RSV promoter and in a non-mutating context. In the upper panel is cDNA from DT40 cells expressing Amrose and control and in the lower panel is cDNA from DT40 cells expressing mRaspberry and control.
(TIF)

Table S1 **Fluorescent lifetimes of the purified proteins eqFP615, eqFP650, and Amrose v0-v4 in PBS.** *λ*_ex_ - excitation wavelength, *λ*_em_ - emmission wavelength, τ_1_ - fluorescence lifetime 1, B_1_ - pre-exponential factor 1, χ^2^ - fit quality parameter.
(TIF)
