# Supplementary figures and images for: Usefulness of a Darwinian System in a Biotechnological Application: Evolution of Optical Window Fluorescent Protein Variants under Selective Pressure
Source: PLoS One. 2014 Sep 5;9(9):e107069. doi: 10.1371/journal.pone.0107069 (PMC4156574; doi:10.1371/journal.pone.0107069)

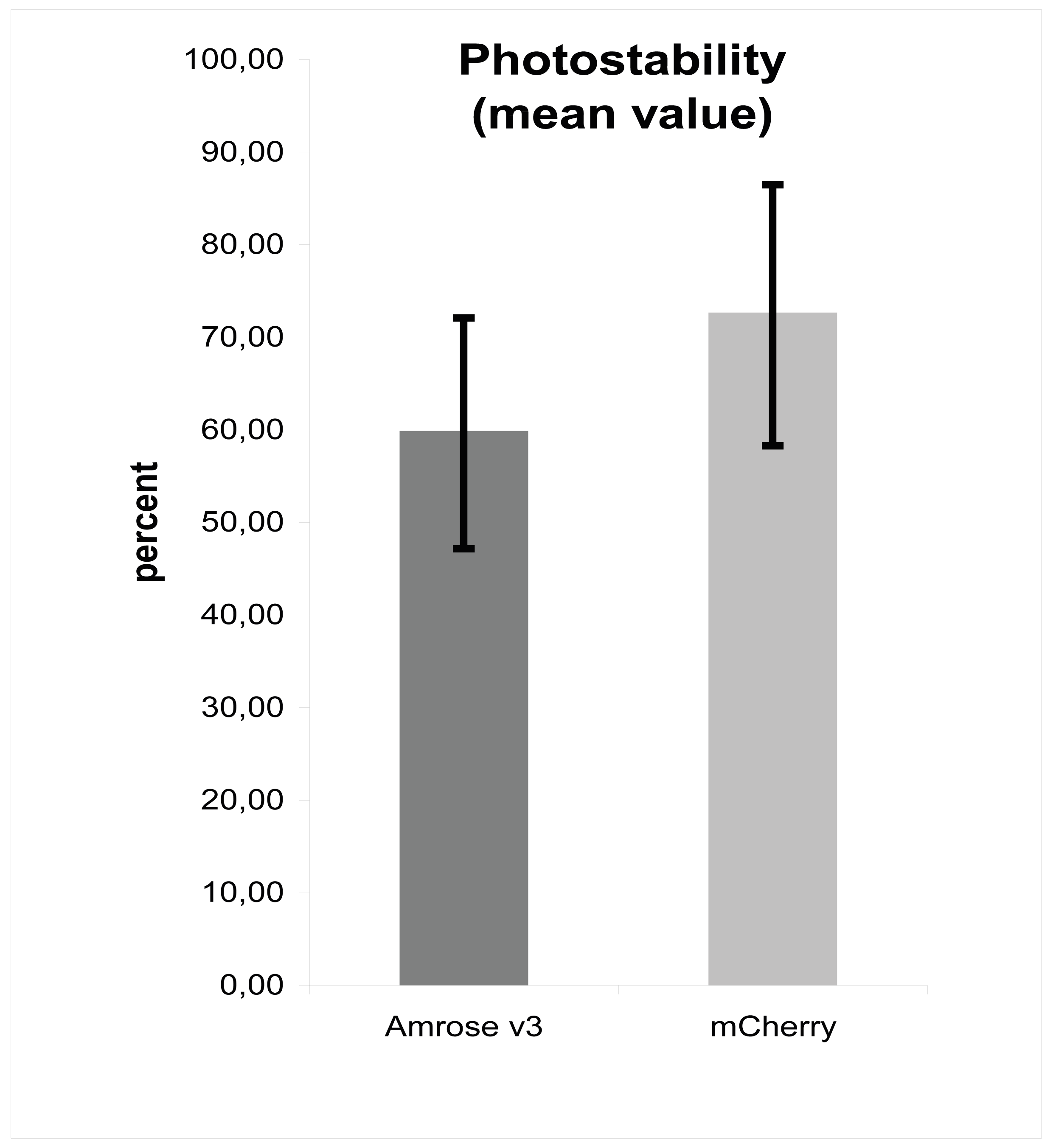

Supplement: Figure S1 — Photostability of Amrose compared to mCherry. Photostability analysis was performed using three zebrafish embryos each for Amrose v3 and mCherry. The embryos were excited using a 633 nm laser set at 100% power and fast scanned producing a series of 150 pictures. The first and last picture of each set measured had ten single corresponding spots compared for loss of emission intensity. (TIF) [file pone.0107069.s001.tif]

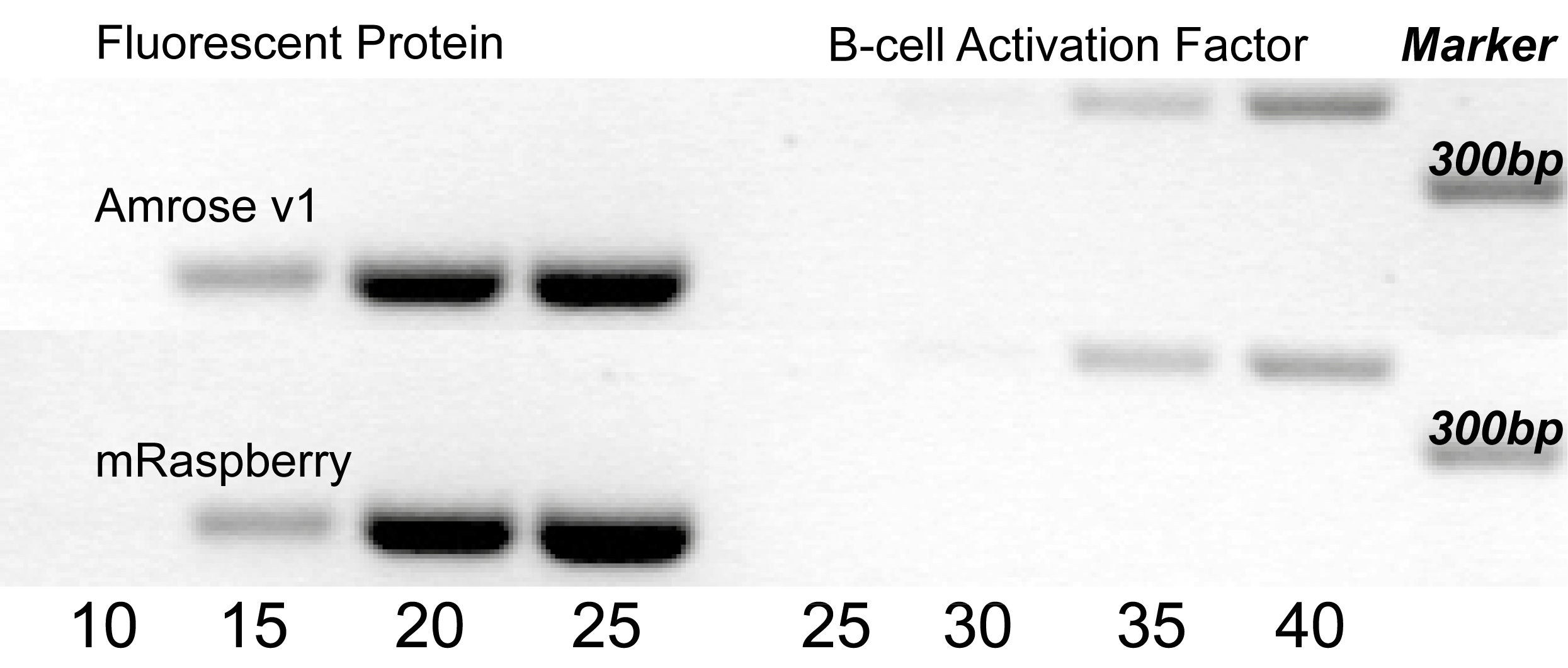

Supplement: Figure S2 — Semi-quantitative expression analysis of Amrose compared to mRaspberry. Amrose and mRaspberry were expressed at comparable levels under the direction of the RSV promoter and in a non-mutating context. In the upper panel is cDNA from DT40 cells expressing Amrose and control and in the lower panel is cDNA from DT40 cells expressing mRaspberry and control. (TIF) [file pone.0107069.s002.tif]

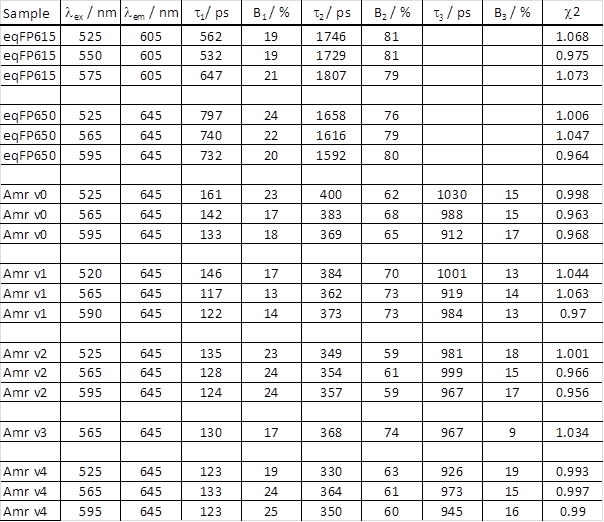

Supplement: Table S1 — Fluorescent lifetimes of the purified proteins eqFP615, eqFP650, and Amrose v0-v4 in PBS. λ ex - excitation wavelength, λ em - emmission wavelength, τ1 - fluorescence lifetime 1, B1 - pre-exponential factor 1, χ2 - fit quality parameter. (TIF) [file pone.0107069.s003.tif]
